# Supplementary figures and images for: Evaluation of Two Real-Time, TaqMan Reverse Transcription-PCR Assays for Detection of Rabies Virus in Circulating Variants from Argentina: Influence of Sequence Variation
Source: Viruses. 2020 Dec 25;13(1):23. doi: 10.3390/v13010023 (PMC7823378; doi:10.3390/v13010023)

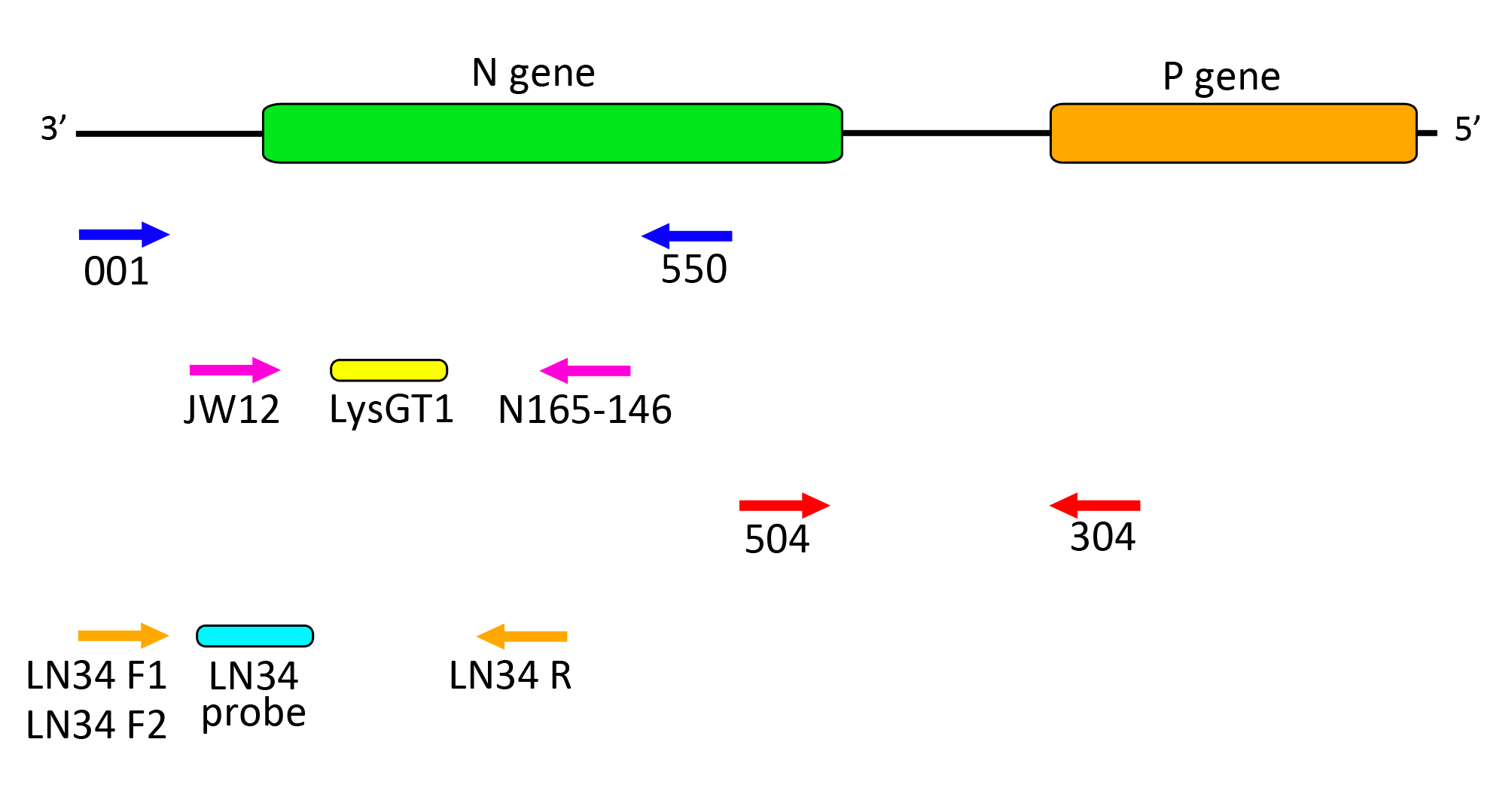

Supplement: Supplementary file 1 [file viruses-13-00023-s001.zip › Supplementary Figure 1.jpg]
